# Long-Term Effects of Pregnancy Complications on Maternal Health: A Review

**DOI:** 10.3390/jcm6080076

**Published:** 2017-07-27

**Authors:** Ran Neiger

**Affiliations:** Director of Maternal-Fetal Medicine Unit, Department of Obstetrics and Gynecology, Ma’ayanei HaYeshua Hospital, 5154475 Bnei Brak, Israel; ran@neiger.com; Tel.: +972-54-5657191

**Keywords:** pregnancy complications, long-term effects, gestational diabetes, preeclampsia, preterm labor

## Abstract

**Background**: Most pregnancy-related medical complications appear to resolve at delivery or shortly thereafter. Common examples are preterm labor, placental abruption, preeclampsia, and gestational diabetes. Women who developed such complications are known to be at increased risk of developing similar complications in future pregnancies. It has recently become evident that these women are at an increased risk of long term medical complications. **Methods**: A search through scientific publications in English regarding the association of obstetric complications and long-term maternal illness. **Results**: There is a clear association between various obstetric complications and long-term effects on maternal health. **Conclusions**: Women with a history of adverse pregnancy outcomes are at increased risk of cardiovascular and metabolic diseases later in life. Data increasingly links maternal vascular, metabolic, and inflammatory complications of pregnancy with an increased risk of vascular disease in later life.

## 1. Introduction

Most pregnancy-related medical complications appear to resolve at delivery or shortly thereafter. Common examples are preterm labor, placental abruption, preeclampsia, and gestational diabetes. Women who developed such complications are known to be at increased risk of developing similar complications in future pregnancies. Women who delivered prematurely are at increased risk of recurrent preterm labor, those who had preeclampsia have an increased risk of preeclampsia in subsequent pregnancies, women who developed gestational diabetes (GDM) are likely to develop it again, as are women who experienced placental abruption, fetal growth impairment, etc. Recently, research has shown that these pregnancy-specific complications continue to affect maternal health long after the index pregnancy. It has become apparent that women with a history of adverse pregnancy outcome are at increased risk of cardiovascular and metabolic diseases later in life. Data increasingly links maternal vascular, metabolic, and inflammatory complications of pregnancy with an increased risk of vascular disease in later life. For example, it has been reported that women who gave birth to very low birthweight babies or experienced combined complications had a several-fold increased risk of mortality from cardiovascular causes [1,2].

Researchers debate whether it was the pregnancy that had this long-term effect on maternal health, or it was the result of predisposing maternal conditions which were expressed during pregnancy and eventually caused chronic morbidity. Researchers who believe the latter coined the phrase, “pregnancy as a stress-test”. In 2005, Sattar and Greer reported on the intriguing probability that complications in pregnancy also predispose mothers to later vascular and metabolic disease [3]. They suggested that pregnancy complications and coronary heart disease may have common disease mechanisms, and that maternal vascular risk factors, potentially “modifiable” before pregnancy, correlated with increased risk of preterm delivery and low birth weight. Similarly, Magnussen et al. hypothesized that cardiovascular risk factors that were present years before pregnancy were associated with increased risk of preeclampsia [4]. Conversely, other researchers associated adverse pregnancy outcome, as well as the increased risk of vascular and metabolic diseases in later life, with placental malfunction, also known as “Placental syndrome”. For example, in 2010 Bronsens et al. [5] suggested that defective deep placentation was associated with a spectrum of pregnancy complications including preeclampsia, intrauterine growth restriction, preterm labor, preterm premature rupture of membranes, late spontaneous abortion, and abruptio placentae. The placental vascular bed disease that underpinned these complications was commonly investigated with targeted biopsies. In their published review, these researchers critically evaluated the biopsy technique to summarize the salient types of defective deep placentation. They proposed criteria for the classification of defective deep placentation into three types, based on the degree of restriction of remodeling and the presence of obstructive lesions in the myometrial segment of the spiral arteries [5]. In 2015 Bronsens et al. suggested that the major obstetric syndromes, including preeclampsia, fetal growth restriction, and spontaneous preterm labor caused by impaired placental bed spiral artery remodeling may be the result of impaired functional maturation of the uterus during the early reproductive years [6]. More recently, Ilekis et al. published a summary of an executive workshop of the Eunice Kennedy Shriver National Institute of Child Health and Human Development on placental origins of adverse pregnancy outcomes—potential molecular targets [7]. According to these researchers, much progress was made in understanding the molecular pathways in the placenta involved in the pathophysiology of pregnancy-related disorders. Utilization of this information could assist in the development of new drug therapies to improve pregnancy outcome, both prevention and treatment of disorders including preeclampsia, fetal growth restriction, and uterine inflammation.

Regardless of whether long-term maternal complications were caused by pregnancy or first recognized during pregnancy, this review summarizes current information regarding the association between the most common obstetric complications—gestational diabetes, preeclampsia, and pre-term labor—and their long term maternal effects. Unfortunately, many obstetricians and gynecologists are not familiar with this information since a great proportion of current research was published in journals other than the “traditional” obstetrics and gynecology literature.

## 2. Gestational Diabetes

When women with history of gestational diabetes (GDM) undergo the 75 gram GTT at 6–12 weeks postpartum, 2–16% are diagnosed with type 2 diabetes (DM) and 36% are found to have intolerance to carbohydrates. Women who had prior GDM have a 36–70% risk of developing type 2 DM later in life, depending on risk factors and length of follow-up. It is important for women who had GDM to have appropriate follow up since, over time, often before patients are diagnosed, DM causes damage to various organs (heart, blood vessels, kidneys, eyes, nerves, etc.) Despite the deceptively benign term, “intolerance to carbohydrates”, this condition is associated with significant morbidity as well. Lee et al. performed a meta-analysis that included 15 prospective studies with 760,925 participants and reported that pre-diabetes, defined as impaired glucose tolerance or a combination of impaired fasting glucose and impaired glucose tolerance, were associated with an increased risk of stroke [8]. Huang et al. performed a meta-analysis of prospective cohort studies to evaluate the associations between pre-diabetes, defined as impaired glucose tolerance, impaired fasting glucose, or raised HbA1c, and the risk of cardiovascular disease and all-cause mortality [9]. Their analysis included 53 prospective cohort studies with 1,611,339 individuals. The median follow-up duration was 9.5 years. Compared with normo-glycaemia, prediabetes was associated with an increased risk of composite cardiovascular disease, stroke, and all-cause mortality. The health risk was increased in people with fasting glucose concentrations as low as 5.6 mmol/L or HbA1c of 39 mmol/mol. Increases in HBA1c to 39–47 were associated with an increased risk of composite cardiovascular disease and coronary heart disease. Follow-up of women who had GDM enables preventive measures and early diagnosis; early detection of DM decreases the risk of complications.

In 2009 Bellamy et al. published a comprehensive systematic review and meta-analysis to assess the strength of association between gestational diabetes and the risk of developing type 2 diabetes and the effect of factors that might modify the risk [10]. They selected 20 studies that included 675,455 women and 10,859 type 2 diabetic events, and calculated and pooled unadjusted relative risks (RRs) with 95% CIs for each study, using a random-effects model. They reported that when compared to women who had a norm-glycemic pregnancy, women who developed gestational diabetes had an increased risk of developing type 2 diabetes (RR 7.43, 95% CI 4.79–11.51). The largest study, that included 659,164 women of which 9502 had type 2 diabetes, had the largest RR (12.6, 95% CI 12.15–13.19). They concluded that increased awareness of the magnitude and timing of the risk of type 2 diabetes after gestational diabetes among patients and clinicians could provide an opportunity to test and use dietary, lifestyle, and pharmacological interventions that might prevent or delay the onset of type 2 diabetes in affected women. Göbl et al. followed women with a history of GDM annually for up to 10 years and reported that impaired glucose tolerance, HDL cholesterol less than 50 mg/dL, and an age older than 35 years were identified as the best predictors of developing diabetes after GDM [11].

Women previously diagnosed with GDM are also at increased risk of future metabolic syndrome, a combination of metabolic abnormalities that include hypertension, DM, dyslipidemia, and obesity, all of which increase the risk of cardiovascular disease. Lauenborg et al. [12] studied the prevalence of metabolic syndrome using three different criteria—World Health Organization 1999 (WHO), The National Cholesterol Education Program Expert Panel on Detection, Evaluation, and Treatment of High Blood Cholesterol in Adults 2001, and European Group for the Study of Insulin Resistance 2002—among 481 Danish women with previous diet-treated GDM. Follow-up occurred at a median of 9.8 years after pregnancy. The prevalence of metabolic syndrome was three times as high in women with prior diet-treated GDM, compared with age-matched control subjects (*p* < 0.0005). Age- and BMI-adjusted odds ratio for having the WHO-defined metabolic syndrome was 3.4 for the prior GDM group vs. the control group. Obese women (BMI > 30 with previous GDM had more than 7-fold increase in prevalence of metabolic syndrome compared with normal-weight prior GDM women (BMI < 25). In glucose-tolerant women, the prevalence was doubled in the prior GDM group, compared with control group [12]. Valizadeh et al. also studied risk factors and incidences of abnormal glucose levels and metabolic syndrome in 110 women with history of GDM one to six years after their pregnancy. Thirty-six women (32.7%) developed type 2 diabetes, 11 (10%) had impaired fasting glucose or impaired glucose tolerance and 22 women (20%) developed metabolic syndrome [13]. The authors suggested that women with a history of GDM should be screened at regular intervals for diabetes and other cardiovascular risk factors.

Gestational diabetes mellitus (GDM) is a risk factor for the development of endothelial dysfunction and cardiovascular disease. It is possible that damage to blood vessels that increases the risk of future cardiovascular disease develops during pregnancy complicated by GDM or even prior to it. In a recent study, 29 pregnant women (13 with GDM and 16 women with uncomplicated pregnancy, at 28 ± 2 gestational weeks) underwent arterial stiffness evaluation. Women with GDM exhibited a characteristic blunted tissue oxygen saturation index curve, showing alterations in muscle oxygenation and microvascular responsiveness compared with women with uncomplicated pregnancies [14]. It is unknown whether hyperglycemia and insulin resistance causes endothelial dysfunction or whether endothelial dysfunction already exists prior to GDM diagnosis and is unmasked by the pregnancy. Regardless, identifying abnormalities in the reactivity of skeletal muscle vessels denotes an increased risk for cardiovascular events, type 2 diabetes, and hypertension in postpartum years. Accordingly, the authors suggested examination of lifestyle and/or pharmacological interventions that would reverse this microvascular dysfunction or differentiate responders from non-responders to therapy. Kessous et al. reported an association between GDM and maternal cardiovascular morbidity [15]. The authors of that population-based study followed a cohort of women with and without a diagnosis of GDM who delivered between the years 1988–1999, with a follow-up period until 2010. Of 47,909 deliveries that met the inclusion criteria, 4928 (10.3%) were women who were diagnosed with GDM. During a follow-up period of over 10 years, compared with women who gave birth at the same time period, after adjustment for age and ethnicity, patients with GDM had higher rates of cardiovascular morbidity including non-invasive cardiac diagnostic procedures (OR = 1.8; 95% CI 1.4 to 2.2), simple cardiovascular events (OR = 2.7; 95% CI 2.4 to 3.1) and total cardiovascular hospitalizations (OR = 2.3; 95% CI 2.0 to 2.5). In a Cox proportional hazards model adjusted for comorbidities such as preeclampsia and obesity, GDM was independently associated with cardiovascular hospitalizations (adjusted HR 2.6, 95% CI 2.3 to 3).

It appears that a high glucose level during pregnancy, even if within the range of slight glucose intolerance, may serve as a marker for future maternal atherosclerotic morbidity. Gunderson et al. measured the common carotid intima media thickness (ccIMT) in 119 women who had a history of GDM and compared it to 779 who did not [16]. Average age was 31 at last birth and 44 at ccIMT measurement for GDM and non-GDM groups. Mean ccIMT was 0.023 mm higher for GDM versus non-GDM groups, controlling for race, age, parity, and pre-pregnancy BMI. Addition of pre-pregnancy insulin resistance index had minimal impact on adjusted mean net ccIMT difference (0.22 mm). Mean ccIMT did not differ by GDM status among 121 women who developed DM or the metabolic syndrome. The authors concluded that a history of GDM may be a marker for early atherosclerosis, independent of pre-pregnancy obesity among women, including those who did not develop type 2 diabetes or the metabolic syndrome. Charach et al. studied 815 women who delivered between the years 2000–2012 who had at least one glucose measurement during pregnancy and subsequently developed atherosclerotic morbidity [17]. They reported a significant linear association between glucose levels during pregnancy and long-term maternal atherosclerotic morbidity. Among cases with severe atherosclerotic morbidity, the proportion of women with a high glucose level (>5.5 mmol/L) was the highest, whereas in 6065 controls it was the lowest (*p* < 0.001). In a Cox proportional hazard model, adjusted for atherosclerotic confounders such as gestational diabetes, preeclampsia and obesity, a glucose level of >5.5 mmol/L was noted as an independent risk factor for hospitalizations later in non-pregnant life (hazard ratio = 1.3, 95% confidence interval 1.1–1.5, *p* < 0.003).

Gestational diabetes is also associated with an increased risk of future renal morbidity. A population-based study compared the incidence of renal morbidity in a cohort of 97,968 women of whom 9542 (9.7%) had at least one previous pregnancy complicated by GDM [18]. Deliveries occurred during a 25-year period, with a mean follow-up duration of 11.2 years. Women with history of GDM had higher rates of renal morbidity. There was a significant dose-response association between the number of pregnancies complicated by GDM and risk of future renal morbidity. Recently, Beharier et al. reported that GDM was also a significant risk factor for long-term ophthalmic morbidity [19]. They compared the incidence of long-term maternal ophthalmic morbidity in a cohort of women with and without a history of GDM. They analyzed 104,751 singleton deliveries; 9.4% (*n* = 9888) of which occurred were in women diagnosed with GDM. Women who had GDM had a significantly higher incidence of ophthalmic morbidity such as glaucoma, diabetic retinopathy, and retinal detachment compared with controls (0.1 vs. 0.02%, *p* < 0.001; 0.2 vs. 0.04%, *p* < 0.001; 0.2 vs. 0.1%, *p* < 0.001, respectively). Women with concurrent GDM and preeclampsia had a significantly higher incidence of ophthalmic complications compared to patients who had only GDM.

In summary, gestational diabetes mellitus (GDM) is an independent risk factor for future type 2 diabetes mellitus, metabolic syndrome, cardiovascular morbidity, vascular endothelial dysfunction, renal and ophthalmic disease. The risk of these conditions may be decreased with proper prevention and interventions. However, the majority of women diagnosed with GDM do not undergo evaluation after pregnancy. According to one study, only 19% (4486 of 23,999) women who had GDM underwent testing to rule out type 2 DM within six months following pregnancy [20]. The authors of another study reported that women who developed GDM rarely followed the recommended dietary and physical activity guidelines in the postpartum period [21]. This is despite the evidence that among women who had GDM, a modest post-partum weight loss resulted in significantly lower increases in fasting glucose and a significant reduction in 2-h glucose, while a 1-kg increase in weight was significantly associated with increase in fasting and 2-h glucose [22]. Ferrara et al. reported that a lifestyle intervention that started during pregnancy and continued postpartum was feasible, prevented pregnancy weight retention, and helped overweight women lose weight [23]. These findings and similar studies were the reason that Gabbe et al. published a “call for action”, an initiative of the National Diabetes Education Program, joined by the American College of Obstetricians and Gynecologists (ACOG) to promote opportunities for obstetrician-gynecologists (ob-gyns) and other primary care providers to better meet the long-term health needs of women with prior gestational diabetes mellitus (GDM), and their children [24]. The authors stated that women with GDM face a lifelong increased risk for subsequent diabetes, primarily type 2 diabetes mellitus. Timely testing for prediabetes may provide an opportunity for ob-gyns to prevent or delay the onset of type 2 diabetes mellitus through diet, physical activity, weight management, and pharmacologic intervention. ACOG and the American Diabetes Association recommend testing women with a history of GDM at 6–12 weeks postpartum. If the postpartum test is normal, they should be retested every three years and at the first prenatal visit in a subsequent pregnancy. If prediabetes is diagnosed, women should be tested annually. Because children of GDM pregnancies face an increased risk for obesity and type 2 diabetes mellitus, families need support to develop healthy eating and physical activity behaviors.

## 3. Preeclampsia

Preeclampsia is one of the leading causes of maternal morbidity and mortality worldwide. It is a pregnancy-specific multi-organ syndrome that affects 2–8% of pregnancies. It is a condition of placental pathogenesis with acute onset of predominantly cardiovascular manifestations attributable to generalized vascular endothelial activation and vasospasm resulting in hypertension and multi-organ hypo-perfusion. It is defined as a new onset of a multisystem pregnancy-related disorder that includes hypertension and either proteinuria or end-organ dysfunction, identified after 20 weeks gestation. During the index pregnancy, preeclampsia causes multi-system damages due to microangiopathy that lead to maternal morbidity that may include cardiac and renal failure, liver damage, cerebrovascular bleeding, pulmonary edema, disseminated intravascular coagulopathy (DIC), placental ischemia, etc. The effects on the fetus include prematurity (due to indicated preterm deliveries), fetal growth impairment, and intrauterine fetal demise. After delivery, the disorder tends to resolve in the majority of women although some remain hypertensive. There is a significant risk of preeclampsia recurrence in future pregnancies. There is an increased lifetime risk of chronic hypertension, cardiovascular disease (CVD), and stroke in women who experienced preeclampsia during pregnancy. The risk is related to the severity of the hypertensive disorder during pregnancy and the gestational age at the time of onset. The terms “preterm” or “early-onset” preeclampsia are used to delineate the severity of the disease in relation to the need for iatrogenic delivery before 37 weeks or the time of the diagnosis at or before 34 weeks of gestational age. Early-onset preeclampsia is especially associated with poor placentation, fetal growth restriction, and worse long-term maternal cardiovascular outcomes than late-onset preeclampsia, whose pathogenesis is more related to predisposing cardiovascular or metabolic risks for endothelial dysfunction.

One of the earliest publications that identified the risk of later-life maternal cardiac disease was published in 1927 by Cowin and Herrick [25]. More recently, Irgens et al. [26] reported that women who had preeclampsia during pregnancy that ended in preterm delivery had an eight fold higher risk of death from CVD compared with women who did not have preeclampsia and delivered at term. The authors studied Norwegian mothers (and fathers) of all 626,272 births that were the mothers' first deliveries, from 1967 to 1992, and divided them into two cohorts based on whether the mother had preeclampsia during the pregnancy. Subjects were also stratified by whether the birth was term or preterm, given that preeclampsia might be more severe in preterm pregnancies. Women who had preeclampsia had a 1.2-fold higher long term risk of death than women who did not have preeclampsia. The risk in women with preeclampsia and a preterm delivery was 2.71-fold higher than in women who did not have preeclampsia, and whose pregnancies went to term. In particular, the risk of death from cardiovascular causes among women with preeclampsia and a preterm delivery was 8.12-fold higher. The authors speculated that genetic factors that increase the risk of cardiovascular disease may also be linked to preeclampsia. Wilson et al. studied the association between hypertensive diseases of pregnancy (gestational hypertension and preeclampsia) and the development of circulatory diseases later in life [27]. They matched a cohort study of 1200 women who had preeclampsia during their first singleton pregnancy with two comparison groups, 1200 with gestational hypertension and 1200 with no history of elevated blood pressure. There were significant positive associations between preeclampsia/eclampsia or gestational hypertension and later hypertension in all measures. The adjusted relative risks varied from 1.13–3.72 for gestational hypertension and 1.40–3.98 for preeclampsia or eclampsia. The adjusted incident rate ratio for death from stroke for the preeclampsia/eclampsia group was 3.59. The authors concluded that later in life hypertensive diseases of pregnancy were associated with diseases related to hypertension, and that greater awareness of this association should lead to earlier diagnosis and improved management, and reduce the morbidity and mortality from such diseases. Bellamy et al. [28] analyzed a dataset of 3,488,160 women, 198,252 of them with a history of preeclampsia and reported that the relative risk of women with a history of preeclampsia for hypertension were 3.70 (2.70 to 5.05) after 14.1 years weighted mean follow-up, for ischemic heart disease 2.16 (1.86 to 2.52) after 11.7 years, for stroke 1.81 (1.45 to 2.27) after 10.4 years, and for venous thromboembolism 1.79 (1.37 to 2.33) after 4.7 years. The relative risk of overall mortality after preeclampsia was 1.49 (1.05 to 2.14) after 14.5 years. The authors suggested that the association between preeclampsia and cardiovascular disease in women might reflect a common cause for these conditions, an effect of preeclampsia on disease development, or both. McDonald et al. conducted a meta-analysis that included five case-control and 10 cohort studies with a total of 116,175 women with, and 2,259,576 women without preeclampsia/eclampsia [29]. Most studies focused on women <56 years of age. Relative to women with uncomplicated pregnancies, women with a history of preeclampsia/eclampsia had an increased risk of subsequent cardiac disease in both the case-control studies (odds ratio 2.47, 95% CI 1.22–5.01) and the cohort studies (relative risk [RR] 2.33, 1.95–2.78), as well as an increased risk of cerebrovascular disease (RR 2.03, 1.54–2.67), peripheral arterial disease (RR 1.87, 0.94–3.73), and cardiovascular mortality (RR 2.29, 1.73–3.04). Meta-regression revealed a graded relationship between the severity of preeclampsia/eclampsia and the risk of cardiac disease (mild: RR 2.00, 1.83– 2.19, moderate: RR 2.99, 2.51–3.58, severe: RR 5.36, 3.96–7.27, *p* < 0.0001). In short, women with a history of preeclampsia/eclampsia were found to have approximately double the risk of early cardiac, cerebrovascular, and peripheral arterial disease, and cardiovascular mortality. In 2004 Haukkamaa et al. studied 141 relatively young (<66 years) parous women with angiographically documented coronary artery disease and reported that history of preeclampsia was an independent risk factor for subsequent coronary artery disease [30]. Smith et al. prospectively assessed physical and biochemical cardiovascular risk markers in a group of women who developed preeclampsia and in a control group at one year postpartum [31]. Markers of cardiovascular disease were different between the groups. Mathematical modeling of cardiovascular event risk suggests that preeclampsia increased the risk of CVD by 2- to 3-fold; the risk was greatest for women with severe preeclampsia. In 2009 Haukkamaa et al. examined whether women with a history of preeclampsia more often showed signs of atherosclerosis compared with two control groups [32]. Women with previous preeclampsia had significantly (*p* = 0.008) more atherosclerotic plaques than the healthy parous controls. The intima-media thickness in women with previous preeclampsia also tended to be higher than in the other groups, although the differences did not reach statistical significance. In logistic regression analysis, advanced age and preeclampsia were independent risk factors for developing atherosclerotic plaques.

Similar to the long-term risks of maternal morbidity associated with other pregnancy complications, e.g., gestational diabetes, it is unknown whether preeclampsia was actually the cause of increased risk of morbidity in these women or merely identified women who had a-priori increased risk of CVD morbidity. Researchers who believe in the former theory coined the expression, “maternal placental syndrome” (MPS), a term that combines various pregnancy complications (e.g., hypertensive disorders, placental abruption, infarction of the placenta, etc.) that originated (in their opinion) from “diseased placental vessels”, often in women who had metabolic risk factors for CVD (including obesity, pre-pregnancy hypertension, diabetes mellitus, and dyslipidaemia). Ray et al. conducted a population-based retrospective cohort study of 1.03 million women, of whom 75,380 (7%) were diagnosed with a maternal placental syndrome, who were free from cardiovascular disease before their first documented delivery, in order to assess the risk of premature vascular disease in women who had had a pregnancy affected by maternal placental syndrome [33]. They defined the following as maternal placental syndromes: preeclampsia, gestational hypertension, placental abruption, and placental infarction. Their primary endpoint was a composite of cardiovascular disease, defined as hospital admission or revascularization for coronary artery, cerebrovascular, or peripheral artery disease at least 90 days after the delivery discharge date. The incidence of cardiovascular disease was 500 per million person-years in women who previously had a maternal placental syndrome compared with 200 per million in women who had not (adjusted hazard ratio [HR] 2.0, 95 CI 1.7–2.2). This risk was higher in the combined presence of a maternal placental syndrome and poor fetal growth (3.1, 2.2–4.5) or a maternal placental syndrome and intrauterine fetal death (4.4, 2.4–7.9). They recommended that women who were affected by maternal placental syndrome should have their blood pressure and weight assessed about six months postpartum, and that a healthy lifestyle should be emphasized. In 2012 Ray et al. studied the risk of new onset of heart failure (HF) and dysrhythmias in 75,242 women whose pregnancy was complicated by MPS, who were among 1,130,764 women who delivered between 1992 and 2009, excluding those with cardiac or thyroid disease one year before delivery. The risk of a composite outcome of a hospitalization for HF or an atrial or ventricular dysrhythmia was compared in women with and without MPS, starting one year after delivery [34]. After a median duration of 7.8 years, the composite outcome occurred in 148 women with MPS (2.54 per 10,000 person-years) and 1062 women without MPS (1.28 per 10,000 person-years) (crude HR = 2.00, 95% CI 1.68 to 2.38). The mean age at composite outcome was 37.8 years. The HR was 1.61 (95% CI 1.35 to 1.91) after adjustment for demographic characteristics, diabetes, obesity, dyslipidaemia and drug dependence or tobacco use, as well as coronary artery disease or thyroid disease >1 year after delivery. The adjusted HRs were minimally reduced by further adjusting for chronic hypertension (1.51, 95% CI 1.26 to 1.80) and were higher in women with MPS plus preterm delivery and poor fetal growth (2.42, 95% CI 1.25 to 4.67). In 2016 Ray et al. reported that a history of maternal placental syndromes increases the risk of death after coronary artery revascularization in middle-aged women [35]. Three hundred and sixty-two of 1,985 women (18.2%) who underwent coronary artery revascularization had a previous maternal placental syndrome event. The mean age at index coronary revascularization was 45 years; percutaneous coronary intervention comprised approximately 80% of procedures. After a mean follow-up time of approximately five years, 41 deaths (2.2 per 100 person-years) occurred in women with previous maternal placental syndromes and 83 deaths (1.1 per 100 person-years) in women without maternal placental syndrome (adjusted hazard ratio, 1.96; 95% confidence interval, 1.29–2.99). Of the maternal placental syndrome subtypes, the risk of death was significant in women with placental abruption (adjusted hazard ratio, 2.79; 95% confidence interval, 1.31–5.96), placental infarction (adjusted hazard ratio, 3.09; 95% confidence interval, 1.23–7.74), and preeclampsia (adjusted hazard ratio, 1.61; 95% confidence interval, 1.00–2.58). Women with maternal placental syndrome in ≥2 pregnancies had the highest adjusted hazard ratio of death (4.31; 95% confidence interval, 1.71–10.89).

Regardless of whether placental pathology causes preeclampsia or preeclampsia and CVD have common causes, it is clear that the diagnosis of preeclampsia denotes an increased risk of future morbidity in previously preeclamptic women. Smith et al. calculated the cardiovascular disease risk estimates at 10-year, 30-year, and lifetime CVD risk at one year postpartum following a pregnancy with or without preeclampsia [36]. A total of 18.2% of preeclamptic women and 1.7% of control women had a high 10-year risk (OR 13.08; 95% CI 3.38 to 85.5), 31.3% of preeclamptic women and 5.1% of control women had a high 30-year risk (OR 8.43; 95% CI 3.48 to 23.23), and 41.4% of preeclamptic women and 17.8% of control women had a high lifetime risk for CVD (OR 3.25; 95% CI 1.76 to 6.11). They concluded that the association of preeclampsia with the future development of CVD made pregnancy an early window of opportunity for the preservation of health and prevention of CVD. A year later, Shalom et al. evaluated the risk of long-term morbidity of patients with history of hypertensive disorders of pregnancy and reported similar findings [37]. They studied 2072 women with history of mild or severe preeclampsia in one or more pregnancies who gave birth between 1988 and 1998, followed until December 2009, and compared them to 20,742 women without preeclampsia. Excluded from the study were patients with chronic hypertension and pre-gestational diabetes before the index pregnancy. Women with history of preeclampsia had significantly higher rates of chronic hypertension (12.5% vs. 0.9%) and were more likely to be hospitalized at least once in either the internal medicine, oncology, nephrology, neurology, cardiac intensive care unit or hematology departments (13.7% vs. 11.4%) compared with women without a history of preeclampsia. More recently, Canoy et al. examined the prospective relation between a history of hypertension during pregnancy and coronary heart disease (CHD) and stroke in middle-aged UK women [38]. Between 1996 and 2001, 1.1 million parous women (mean age = 56 years) without vascular disease at baseline reported their history of hypertension during pregnancy and other factors. They were followed for incident CHD and stroke (hospitalization or death). Twenty-six percent (290,008/1.1 million) reported having had a hypertensive pregnancy; 27% (79,163/290,008) of women with hypertensive pregnancy, but only 10% (82,145/815,560) of those without hypertensive pregnancy, reported being treated for hypertension at baseline. Mean follow-up was 11.6 years (mean ages at diagnosis/*N* of events: CHD = 65 years/*N* = 68,161, ischemic stroke = 67 years/*N* = 8365, hemorrhagic stroke = 64 years/*N* = 5702). Overall, the RRs of incident disease in women with hypertensive pregnancy versus those without such history were: CHD = 1.29 (1.27–1.31), ischemic stroke = 1.29 (1.23–1.35), and hemorrhagic stroke = 1.14 (1.07–1.21). However, among women with hypertensive pregnancy who were not taking hypertension treatment at baseline, their RRs were only modestly increased: CHD = 1.17 (1.14–1.19), ischemic stroke = 1.18 (1.11–1.25), and hemorrhagic stroke = 1.09 (1.02–1.18). The authors concluded that hypertension during pregnancy was associated with increased CHD and stroke incidence in middle age, largely because such women also had hypertension in their 50s and 60s, which has a substantially greater effect on vascular disease risk than hypertension during pregnancy without hypertension later in life. In 2017 Auger et al. studied the association between recurrent preeclampsia and long-term cardiovascular hospitalization [39]. They identified cardiovascular hospitalizations up to 25 years after pregnancy for all women who delivered between 1989 and 2013 in Québec, Canada. Exposures included recurrent and non-recurrent preeclampsia in women with two deliveries or more (*N* = 606,820), and preeclampsia in women with only one delivery (*N* = 501,761). Incidence, timing, and risk of cardiovascular complications were calculated using accelerated failure time models adjusted for age, pre-existing disease, socioeconomic deprivation and period. Outcomes included a range of cardiovascular hospitalizations and procedures. They reported that women with recurrent preeclampsia had higher incidence of cardiovascular hospitalization (281.4 per 1000) than women with non-recurrent (167.7 per 1000) or no preeclampsia (72.6 per 1000). Mean time of cardiovascular hospitalization was 10.5 years for recurrent, 11.6 years for non-recurrent, and 12.7 years for no preeclampsia, a difference of 17.3% for recurrent and 8.7% for non-recurrent relative to no preeclampsia. Compared with no preeclampsia, recurrent preeclampsia was associated with twice the risk of heart disease (95% CI 1.69 to 2.29) and three times the risk of cerebrovascular disease (95% CI 2.25 to 4.05). Preeclampsia in women with one delivery was associated with three times greater risk of cardiovascular hospitalization compared with no preeclampsia in women with two deliveries or more. Bosklag et al. assessed the prevalence of risk factors or actual signs of cardiovascular disease 5–20 years after pregnancies complicated by early-onset preeclampsia [40]. They felt that the presence of hypertension or metabolic syndrome could be seen as an opportunity for preventive interventions to reduce the development of severe cardiovascular diseases like myocardial infarction and stroke. In a prospective observational study, cardiovascular risk assessment was performed in 131 women with early-onset preeclampsia (<34 weeks’ gestation) and 56 normotensive controls (≥37 weeks’ gestation) 9–16 years after their index pregnancy. Women with a history of early-onset preeclampsia had significantly greater systolic and diastolic blood pressure, greater body mass index, more often had an abnormal lipid profile (lower high-density lipoprotein levels, higher triglycerides), greater glycated hemoglobin, and greater levels of albuminuria compared to controls. None of the women with a history of early-onset preeclampsia were diagnosed with cardiovascular disease; 38.2% were diagnosed with hypertension; and 18.2% were diagnosed with metabolic syndrome. In women with a history of uncomplicated pregnancy, no women were diagnosed with cardiovascular disease; 14.3% were diagnosed with hypertension; 1.8% with metabolic syndrome. The authors concluded that a large proportion of women who experienced early-onset preeclampsia had major cardiovascular risk factors in the fifth decade of life, compared with healthy controls. These women were outside the scope of most preventive programs due to their relatively young age, but had important modifiable risk factors for cardiovascular diseases.

The cause of the association between preeclampsia and future risk of CVD is unknown. McDonald et al. investigated whether the increased risk of CVD in formerly preeclamptic women was related to albuminuria, a known cardiovascular risk factor that is part of the definition of preeclampsia and that often persists after delivery [41]. They performed a cross-sectional analysis of 4080 dysglycemic women enrolled in a large randomized controlled trial who provided an obstetric history and had at least one delivery. There were 3613 women with no history of preeclampsia during their pregnancies, 108 with severe preeclampsia, and 359 with non-severe preeclampsia. Women with a history of severe preeclampsia had higher rates of previous cardiovascular disease than women with non-severe preeclampsia or women without preeclampsia (87, 72 and 72%, *p* = 0.0019). The high risk of previous cardiovascular disease in women with a history of severe preeclampsia persisted after adjustment for albuminuria and also after adjusting for other covariates, including albuminuria. The author concluded that even after accounting for cardiovascular risk factors including albuminuria, a history of severe preeclampsia was independently associated with a threefold higher risk of cardiovascular disease. Formerly preeclamptic women have an increased risk of elevated c-reactive protein (CRP). Elevated CRP levels have been associated with hypertension in pregnancy and with CVD. Brown et al. assessed whether hypertension in pregnancy was associated with elevated CRP levels in later life, possibly reflecting an increased risk of CVD [42]. They studied 2463 women from the Genetic Epidemiology Network of Arteriopathy (GENOA) study. Participants were categorized as nulliparous women (*n* = 219), women with a history of normotensive pregnancies (*n* = 1839), or women with a history of a hypertensive pregnancy (*n* = 405). Using multiple linear regression models, they compared mean CRP levels among the groups after adjusting for age, race, education, smoking, hypertension, personal history of coronary heart disease (CHD) or stroke, diabetes, dyslipidemia, statins, hormone replacement therapy, and family history of CHD or stroke. As CRP levels may be influenced by BMI, the model was fit both with and without adjusting for BMI. There was no significant difference in CRP levels between nulliparous women and those with a history of normotensive pregnancies, either with (*p* = 0.82) or without (*p* = 0.46) adjusting for BMI. In contrast, women with hypertensive pregnancies, compared with those with normotensive pregnancies, had higher CRP levels, both with (*p* = 0.009) and without (*p* < 0.001) adjusting for BMI. The authors concluded that a history of hypertension in pregnancy is associated with elevated CRP levels later in life, independent of traditional CVD risk factors and BMI. An elevated CRP might reflect an inflammatory state in women with a history of hypertensive pregnancy disorders who were at increased risk for CVD. A study published in 2016 by Weissgerber et al. sought to isolate the effect of hypertension in pregnancy by comparing the risk of hypertension and cardiovascular disease in women who had hypertension in pregnancy and their sisters who did not, using the dataset from the Genetic Epidemiology Network of Arteriopathy study which examined the genetics of hypertension in white, black, and Hispanic siblings [43]. They included all sib-ships with at least one parous woman and at least one other sibling. Compared with their sisters who did not have hypertension in pregnancy, women who had hypertension in pregnancy were more likely to develop new onset hypertension later in life, after adjusting for body mass index and diabetes (hazard ratio 1.75, 95% confidence interval 1.27–2.42). A sibling history of hypertension in pregnancy was also associated with an increased risk of hypertension in brothers and unaffected sisters, whereas an increased risk of cardiovascular events was observed in brothers only. These results suggested that familial factors contribute to the increased risk of future hypertension in women who had hypertension in pregnancy.

In 2013 Brown et al. published a review and meta-analysis in order to assess and quantify the risks of cardiovascular disease (CVD), cerebrovascular events, and hypertension associated with prior diagnosis of preeclampsia [44]. Fifty articles were included in the review, and 43 in the meta-analysis. Women with a history of preeclampsia or eclampsia were at significantly increased odds of fatal or diagnosed CVD (odds ratio (OR) = 2.28, 95% confidence interval (CI: 1.87, 2.78)), cerebrovascular disease (OR = 1.76, 95% CI 1.43, 2.21) and hypertension (relative risk (RR) = 3.13, 95% CI 2.51, 3.89). They concluded that women diagnosed with history of preeclampsia were at increased risk of future cardiovascular or cerebrovascular events, with an estimated doubling of odds compared to unaffected women. This association might reflect shared common risk factors for both preeclampsia and cardiovascular and cerebrovascular disease. In 2014 Melchiorre et al. published an overview of cardiovascular implications of preeclampsia [45]. In their discussion regarding the cardiovascular system in postpartum recovery from preeclampsia, the authors reported that changes in both arterial and venous systems seen in acute preeclampsia persist for the first few years postpartum and were associated with abnormal autonomic response to volume expansion and exercise. Asymptomatic left ventricular (LV) systolic and diastolic dysfunction, LV hypertrophy, and a pre-hypertension state also persisted at one year postpartum, and were more prevalent in women who experienced preterm preeclampsia (60%) compared with term preeclampsia (15%) or matched controls (8%). More than half of preterm women with preeclampsia had asymptomatic LV cardiac dysfunction or hypertrophy (stage B heart failure) postpartum, and 40% developed essential hypertension within 1–2 years after pregnancy. The relative risk of developing hypertension within two years of birth, even after adjusting for confounding risk factors, was increased 15-fold if LV abnormalities persisted. The higher prevalence of stage B heart failure in preterm than in term preeclampsia or controls was consistent with the long-term outcome studies, demonstrating that women with preterm preeclampsia had a higher risk of subsequent congestive heart failure and ischemic cardiac diseases compared with women with term preeclampsia or normal pregnancy. In 2015 Veerbeek et al. also reported that postpartum there were differences in the prevalence of common modifiable CVD risk factors between women who had preterm and term preeclampsia [46]. These researchers compared the prevalence of common CVD risk factors postpartum among 448 women with previous early-onset preeclampsia, 76 women with previous late-onset preeclampsia, and 224 women with previous pregnancy-induced hypertension. Compared to women with late-onset preeclampsia and pregnancy-induced hypertension, women with previous early-onset preeclampsia had significantly higher fasting blood glucose (5.29 versus 4.80 and 4.83 mmol/L), insulin (9.12 versus 6.31 and 6.7 uIU/L), triglycerides (1.32 versus 1.02 and 0.97 mmol/L), and total cholesterol (5.14 versus 4.73 and 4.73 mmol/L). Almost half of the early-onset preeclampsia women had developed hypertension, as opposed to 39% and 25% of women in the pregnancy-induced hypertension and late-onset preeclampsia groups, respectively. The authors suggested that prevention strategies should be stratified according to severity and gestational age of onset of hypertensive disorders of pregnancy.

History of preeclampsia denotes risks of morbidity in other organ-systems, as well. Aukes et al. reported that history of preeclampsia was a risk marker for early cerebrovascular damage [47]. They noted that formerly eclamptic women demonstrate cerebral white matter lesions (WMLs) several years following the index pregnancy. The pathophysiology might be related to the predisposition for cerebrovascular/cardiovascular disease in such women and/or the occurrence of posterior reversible encephalopathy syndrome while pregnant. They performed cerebral magnetic resonance imaging scans in 73 formerly preeclamptic women, matched them with parous control women, and reported that formerly preeclamptic women had white matter lesions significantly more often (37%) and more severely than controls (21%, *p* = 0.04). Weigman et al. also noted that complete neurocognitive recovery after eclampsia had been questioned with the expression of neurocognitive deficits by affected women and demonstration of cerebral white matter lesions on magnetic resonance imaging years after eclampsia [48]. They studied the vision-related quality of life (QOL) in 47 formerly eclamptic women and 47 control participants 10.1 ± 5.2 and 11.5 ± 7.8 years after their index pregnancy, respectively. Composite scores were significantly lower in formerly eclamptic women than in control participants (*p* < 0.01 for composite scores). This could not be explained by visual field loss, because all formerly eclamptic women who underwent perimetry (*n* = 43) demonstrated intact visual fields. White matter lesions were present in 35.7% of formerly eclamptic women who underwent magnetic resonance imaging (*n* = 42) and were associated with lower vision-related QOL scores (*p* < 0.05 for composite scores. Beharier et al. also investigated whether patients with history of preeclampsia had an increased risk of long-term ophthalmic complications [49]. Of 103,183 deliveries, there were 8324 (8.1%) complicated by preeclampsia. Women who had preeclampsia had a significantly higher incidence of long-term ophthalmic morbidity such as diabetic retinopathy and retinal detachment. In addition, a positive linear correlation was found between the severity of preeclampsia and the prevalence of future ophthalmic morbidities (0.3 vs. 0.5 vs. 2.2%, respectively). Kaplan-Meier survival curve indicated that women with preeclampsia had higher rates of total ophthalmic morbidity (0.2 vs. 0.4%, for no preeclampsia and with preeclampsia, respectively; odds ratio = 2.06, 95% confidence interval: 1.42–2.99; *p* < 0.001). In a Cox proportional hazards model, adjusted for confounders, a history of preeclampsia remained independently associated with ophthalmic complications. Postman et al. reported that women who experienced preeclampsia more frequently reported daily cognitive failures and showed increased emotional dysfunction several years later, but were not impaired on objective neurocognitive testing [50]. In addition, women with preterm preeclampsia more often had cerebral white matter lesions (WML) on follow-up. These researchers attempted to determine whether WML presence was related to cognitive dysfunction, anxiety, and depressive symptoms in formally preeclamptic women. Forty-one formerly eclamptic, 49 preeclamptic, and 47 control women who had a normotensive pregnancy completed the Cognitive Failures Questionnaire (CFQ), the Hospital Anxiety and Depression Scale (HADS), and a broad neurocognitive test battery (visual perception and speed of information processing, motor functions, working memory, long-term memory, attention, and executive functioning). All underwent cerebral magnetic resonance imaging (MRI), and WML presence was recorded. Median elapsed time since index pregnancy was six years. Average age was 40 years. WML were more prevalent in women who had experienced preterm preeclampsia (<37 weeks; 40%) than in controls (21%, *p* = 0.03). In preeclamptic women, CFQ and HADS scores were higher than those in controls (44 ± 16.1 vs. 36 ± 11.0, *p* < 0.001, and 11 ± 6.3 vs. 8 ± 5.5, *p* < 0.001). There was no difference in objective cognitive performance as measured by neurocognitive tests. Subjective and objective cognitive functioning, anxiety, and depressive symptoms were not related to WML presence. They concluded that formerly preeclamptic women reported cognitive dysfunction, but did not exhibit overt cognitive impairment when objectively tested on average six years following their pregnancy. The presence of WML was not related to objective nor to subjective cognitive impairment, anxiety, and depressive symptoms.

History of preeclampsia is associated with an increased risk of long-term maternal atherosclerotic morbidity. In 2015 Kessous et al. compared the incidence of long-term atherosclerotic morbidity in a cohort of 96,370 women who delivered between 1988 and 2012 [51]. Mean follow-up duration was 11.2 years. A number of 7824 (8.1%) women were diagnosed at least once with preeclampsia. Patients with preeclampsia had higher rates of cardiovascular morbidity including cardiac non-invasive (OR 1.4; *p* = 0.005) and invasive diagnostic procedures (OR 1.7; *p* = 0.001), simple (OR 1.5; *p* = 0.001), as well as complex cardiovascular events (OR 2.4; *p* = 0.001) and renal (OR 3.7; *p* = 0.001) hospitalizations. A significant linear association was noted between the severity of preeclampsia (no preeclampsia, mild preeclampsia, severe preeclampsia, and eclampsia) and cardiovascular (2.7% vs. 4.5% vs. 5.2% vs. 5.7%, respectively; *p* = 0.001), as well as renal disease (0.1% vs. 0.2% vs. 0.5% vs. 1.1%, respectively; *p* = 0.001). Likewise, a linear association was found between the number of previous pregnancies with preeclampsia (no preeclampsia, one event and ≥2 events of preeclampsia) and risk for future simple cardiovascular disease (1.2% vs. 1.6% vs. 2.2%, respectively; *p* = 0.001), complex cardiovascular disease (1.3% vs. 2.7% vs. 4.6%, respectively; *p* = 0.001) and total cardiovascular hospitalizations (2.7% vs. 4.4% vs. 6.0%, respectively; *p* = 0.001). Using a Kaplan-Meier survival curve, patients with preeclampsia had significantly higher cumulative incidence of atherosclerotic-related hospitalizations. In a Cox proportional hazards model, adjusted for confounders such as maternal age, parity, diabetes mellitus, and obesity, preeclampsia remained independently associated with atherosclerotic hospitalizations. Preeclampsia is also associated with an increased risk of cardiomyopathy. Women with hypertensive disorders of pregnancy, and preeclampsia in particular, have an increased risk of cardiomyopathy during the peripartum period. Behrens et al. reported that women with a history of hypertensive disorders of pregnancy, compared with women without such a history, had a small but statistically significant increased risk of cardiomyopathy more than five months after delivery [52]. These researchers compared rates of cardiomyopathy in women with and without a history of hypertensive disorders of pregnancy in a cohort of 1,075,763 women with at least one pregnancy ending in live birth or stillbirth in Denmark, 1978–2012, with follow-up through 31 December, 2012. The women in the primary cohort had 2,067,633 eligible pregnancies during the study period, 76,108 of which were complicated by a hypertensive disorder of pregnancy. During follow-up, 1,577 women (mean age, 48.5 years at cardiomyopathy diagnosis; 2.6% with multiple pregnancies) developed cardiomyopathy. Compared with women with normotensive pregnancies (18,211,603 person-years of follow-up; *n* = 1408 cardiomyopathy events, 7.7/100,000 person-years (95% CI, 7.3–8.2)), women with a history of hypertensive disorders of pregnancy had significantly increased rates of cardiomyopathy. These increases persisted more than five years after the latest pregnancy. Mediation analyses suggested that only about 50% of the association was an indirect association through post-pregnancy chronic hypertension. In this cohort, 11% of all cardiomyopathy events occurred in women with a history of hypertensive disorders of pregnancy. Preeclampsia is associated with an increased risk of prehypertension (preHTN) and hypertension (HTN) in the early years after delivery. Black et al. studied 5,960 women who were normotensive prior to pregnancy; 358 (6.0%) developed a hypertensive disorder in pregnancy, of whom 215 (60.1%) had preeclampsia or eclampsia [53]. Overall, 63 (1.1%) developed HTN and 902 (15.1%) preHTN in the year after delivery. After accounting for all potential confounders, women with a hypertensive disorder in pregnancy and those with preeclampsia/eclampsia were 2.36 and 2.48 times as likely, respectively, to develop preHTN/HTN in the year after delivery as those without pregnancy-related HTN. History of preeclampsia is also associated with an increased risk of future metabolic syndrome. Stekkinger et al. assessed the prevalence of the metabolic syndrome postpartum in women with a history of pregnancy complicated by early-onset vascular disorders compared with women with late-onset disorders [54]. Eight hundred and forty nine women with a history of pregnancy complicated by vascular disorders (preeclampsia; gestational hypertension; hemolysis, elevated liver enzymes, low platelets syndrome; eclampsia; placental abruption; fetal growth restriction; and stillbirth as a result of placental insufficiency) were divided into early-onset (delivery before 32 weeks of gestation, *n* = 376) and late-onset (delivery at or beyond 32 weeks, *n* = 473). The metabolic syndrome was present in 15–25% of women after early-onset vascular-complicated pregnancy and in 10–14% of women after late-onset disease.

Low birthweight is associated with increased rates of coronary heart disease, stroke, hypertension, and non-insulin-dependent diabetes during adult life. It is probably the consequence of a “programming”, whereby a stimulus or insult at a critical, sensitive period of intrauterine life has permanent effects on structure, physiology, and metabolism. Mal-programming of the fetus may result from adaptations to a condition where placental nutrient supply fails to match fetal demand. In 2012 Tranquilli et al. reported that having had preeclampsia is a risk factor not only for the mother’s future health, but also affected the offspring’s adult health [55]. Children born from preeclamptic pregnancies were more prone to hypertension, insulin resistance, and diabetes mellitus, neurological problems, stroke, and mental disorders during their lifetime. Pisaneschi et al. suggested that compensatory feto-placental up-regulation of the nitric oxide system occurred during fetal growth restriction (FGR) [56]. Restricted hypoxic fetuses present an elevation of nitrites and a reduction of asymmetric dimethylarginine. S-nitrosohemoglobin is consumed under hypoxic conditions. These events are followed by nitric oxide pathway down-regulation postnatally, increasing susceptibility to cardiovascular disorders later in life. The relative hyperoxia would favor any such occurrence through depletion of tetrahydrobiopterin secondary to oxygen radical formation. This concept may lead to new therapeutic strategies, based on tetrahydrobiopterin supplementation, free-radical scavenging, L-arginine administration and/or inhaled NO therapy in FGR hypoxic newborns, to improve their postnatal adaptation and to reduce the risk of metabolic pathologies in adult age. In 2013 Leeson wrote that offspring born to women with preeclampsia were also more likely to have a higher blood pressure from childhood and stroke in later life [57]. Furthermore, the risk to mother and offspring was greatest when preeclampsia was diagnosed at an earlier gestation, suggesting a more severe form of preeclampsia. As the long term cardiovascular risk to both mother and child was known from delivery, there was increasing interest in key phenotypic variations that were identifiable in mothers and children during the years between the episode of preeclampsia and the emergence of established cardiovascular disease. These might help explain the link between the two conditions, provide a means to identify subjects at greatest risk of later cardiovascular disease, and establish intermediate endpoints for future preventative interventions. This increased understanding would allow both better characterization of long term cardiovascular outcomes and better identification of optimal approaches to improve long term outcomes.

In 2016 Cain et al. studied the short-term risk (within five years of first pregnancy) of cardiovascular disease among women who experienced maternal placental syndrome, as well as preterm birth and/or delivery of small-for-gestational-age infants [58]. The risk of subsequent cardiovascular disease was compared among women who did and did not experience placental syndrome during their first pregnancy. Risk was then reassessed among women with placental syndrome and preterm birth or delivering a small-for-gestational-age infant vs. those without these adverse pregnancy outcomes. The study population was 302,686 women. Median follow-up time was 4.9 years. The unadjusted rate of subsequent cardiovascular disease among women with any placental syndrome (11.8 per 1000 women) was 39% higher than the rate among women without placental syndrome (8.5 per 1000 women). Even after adjusting for sociodemographic factors, preexisting conditions and clinical and behavioral conditions associated with the current pregnancy, women with any placental syndrome experienced a 19% increased risk of cardiovascular disease. Women with >1 placental syndrome had the highest cardiovascular disease risk (hazard ratio, 1.43; 95% confidence interval, 1.20–1.70), followed by those with eclampsia/preeclampsia alone (hazard ratio, 1.42; 95% confidence interval, 1.14–1.76). When placental syndrome was combined with preterm birth and/or small for gestational age, the adjusted risk of cardiovascular disease increased 45%. Women with placental syndrome who then developed cardiovascular disease experienced a 5-fold increase in health care-related costs during follow-up, compared to those who did not develop cardiovascular disease. The researchers suggested that strategies to identify and lower cardiovascular disease risk in the postpartum period (e.g., earlier lifestyle modifications and disease prevention) might improve future heart disease outcomes. Identification and implementation of interventions in the short term might decrease morbidity in subsequent pregnancies. Does intervention prevent or decrease the risk of morbidity in women who experienced preeclampsia? Research shows it does. Berks et al. assessed whether offering lifestyle intervention after a complicated pregnancy significantly reduced weight and/or other cardiovascular and metabolic risk factors [59]. During the study 1121 women gave birth after a complicated pregnancy. Four hundred and ninety women were eligible for the study, of which 240 women (49%) gave informed consent to participate. Fifty six women (23%) were lost-to-follow-up, leaving 186 women for the analysis. Between six and 13 months postpartum, weight was significantly reduced in cases compared to controls by 2.1 kg (95% >CI 0.4–3.7), resulting in lower BMI. Systolic blood pressure (5.0 mmHg), waist circumference and waist-to-hip ratio were significantly improved compared to controls. Heart rate, hip circumference, and total cholesterol were significantly improved within cases, but not compared to controls. Diastolic blood pressure and fasting glucose were not improved.

In order to decrease the risks of long-term effects on the health of women with history of preeclampsia, women with history of preeclampsia and the medical staff caring for them during the following years need to be familiar with these risks and act to modify them. Taylor et al. studied women’s risk perception for future cardiovascular disease (CVD) after being diagnosed with a hypertensive disorder of pregnancy [60]. Of the 146 subjects included, 28% were diagnosed with preeclampsia with severe features, 52.1% with preeclampsia with mild features, and 19.9% had chronic hypertension. Women with severe features and those delivering preterm were more likely to report a perception of increased risk of both recurrent hypertensive disorders in a future pregnancy (*p* = 0.004 and 0.005, respectively) and hypertension later in life (*p* = 0.01 and 0.03, respectively). Women delivering preterm were more likely to report an accurate perception of increased risk of myocardial infarction and stroke compared to those delivering at term (*p* = 0.006 and 0.002, respectively). The authors concluded that interventions targeted at improved health awareness in women diagnosed with hypertensive disorders of pregnancy were warranted. Heidrich et al. assessed the knowledge of obstetrician-gynecologists in German outpatient care setting regarding the future health risk of preeclampsia and knowledge of the current guidelines on treatment and counseling patients post preeclampsia [61]. A random sample of 500 obstetrician-gynecologists in the federal state of Lower Saxony was mailed a survey and 212 obstetrician-gynecologists (42.4%) responded to the questionnaire. A large proportion of physicians stated that preeclampsia was associated with a higher risk for the development of hypertension (86.6%), stroke (78.5%), and kidney disease (78.0%). Of the participants, 75.8% reported that women with history of preeclampsia had a shorter life expectancy. Respondents with knowledge of the current guidelines of the German Association of Obstetrics and Gynecology concerning follow up and risk management of preeclampsia (45.2%) were more often aware of the development of CVD and stroke, and counseled patients on self-blood-pressure measurement, meaning, and long-term-risks of PE and attached importance to family history of PE, compared to physicians with no knowledge of the guidelines. The authors concluded that although the majority of obstetrician-gynecologists were aware of higher CVD risk after preeclampsia, weaknesses existed in the follow-up care and counseling of these patients. These deficiencies would be amendable to directed educational activities to improve the implementation of current guidelines.

## 4. Preterm Deliveries

Women who delivered prematurely are also at increased risk of long-term cardiovascular disease (CVD) and additional morbidities. Kessous et al. compared the incidence of cardiovascular morbidity in a cohort of 47,908 women, 5992 of whom (12.5%) delivered prematurely (<37 weeks’ gestation), between 1988–1999 with follow-up until 2010 [62]. Women who delivered prematurely (PTD) had higher rates of simple and complex cardiovascular events and higher rates of total cardiovascular-related hospitalizations. A linear association was found between the number of previous PTD and future risk of cardiovascular hospitalizations (5.5% for ≥2 PTDs; 5.0% for 1 PTD vs. 3.5% in the control group; *p* < 0.001). The association remained significant for spontaneous vs. induced PTD and for early (<34 weeks) and late (34 weeks to 36 weeks six days’ gestation) PTD. In a Cox proportional hazards model adjusted for pregnancy confounders such as labor induction, diabetes mellitus, preeclampsia, and obesity, PTD was independently associated with cardiovascular hospitalizations (adjusted hazard ratio, 1.4; 95% confidence interval, 1.2–1.6). In 2014 Robbins et al. summarized 10 studies that assessed the association between having a history of PTB and subsequent CVD morbidity or death [63]. Compared with women who had term deliveries, women with any history of PTB had increased risk of CVD morbidity (variously defined; adjusted hazard ratio [aHR] ranged from 1.2–2.9; 2 studies), ischemic heart disease (aHR, 1.3–2.1; 3 studies), stroke (aHR, 1.7; 1 study), and atherosclerosis (aHR, 4.1; 1 study). Four of five studies that examined death showed that women with a history of PTB have twice the risk of CVD death compared with women who had term births. Two studies reported statistically significant higher risk of CVD-related morbidity and death outcomes (variously defined) among women with ≥2 pregnancies that ended in PTBs compared with women who had at least two births but which ended in only one PTB. Ngo et al. studied 797,056 women who delivered a singleton infant between 1994 and 2011 in New South Wales, Australia in order to determine whether the increased future risk of maternal cardiovascular disease (CVD) associated with preterm birth was independent of maternal smoking during pregnancy [64]. Preterm births were categorized as late (35–36 weeks), moderate (33–34 weeks), or extreme (≤32 weeks); and as spontaneous or indicated. During the study period, 59,563 women (7.5%) had at least one preterm birth. After adjustment for CVD risk factors other than smoking, adjusted hazard ratios (AHR) of CVD among women who ever had a preterm birth was 1.78 [1.61–1.96]. Associations were greater for extreme (AHR = 1.98 [1.63–2.42]) and moderate (AHR = 2.06 [1.69–2.51]) than late preterm birth (AHR = 1.63 [1.44–1.85]), for indicated (AHR = 2.04 [1.75–2.38]) than spontaneous preterm birth (AHR = 1.65 [1.47–1.86]), and for having ≥two (AHR = 2.29 [1.75–2.99]) than having one preterm birth (AHR = 1.73 [1.57–1.92]). A further adjustment for maternal smoking attenuated, but did not eliminate the associations. Smoking during pregnancy was also independently associated with maternal CVD risks. In 2016 Catov et al. reported that women with a history of preterm birth had increased risk of metabolic syndrome compared with those who delivered at term, independent of the pre-pregnancy metabolic status and pregnancy complications [65]. These researchers conducted a multicenter, longitudinal, observational study of women who had at least one birth and no metabolic syndrome or diabetes before pregnancy. Cardio-metabolic factors were measured pre-pregnancy and at up to five subsequent examinations. They estimated the relative hazards of incident metabolic syndrome in 295 women with one or more preterm births compared with 910 women who delivered at term. Of 315 cases of metabolic syndrome in 17,717 person-years of follow-up, the incidence rate was higher among women with preterm compared with term births (22.0 compared with 16.4 per 1000 person-years; relative hazard 2.91 (95% confidence interval (CI) 2.75–3.09)). After adjustment for pre-pregnancy cardio-metabolic factors and covariates, the relative hazard (95% CI) for metabolic syndrome was 1.52 (1.22–1.88) for women with preterm compared with term births. Gestational diabetes mellitus, hypertension during pregnancy, and weight gain only modestly attenuated this association. Elevated blood pressure (36.3% compared with 26.7%, *p* = 0.002) and central adiposity (51.5% compared with 44.0%, *p* = 0.02) were the individual metabolic syndrome components that were different in women with preterm compared with term births. Pariente et al. reported that women who had a preterm delivery (PTD) were at an increased risk of subsequent long term maternal kidney disease [66]. They analyzed 99,338 women who delivered over a period of 25 years, of whom 16,364 (16.4%) had at least one PTD, over a mean follow-up duration of 11.2 years. They reported a significant dose response between the number of previous PTDs and the gestational age at birth of the PTDs and future risk of renal-related hospitalizations. Women with either spontaneous or indicated PTD had higher rates of renal-related hospitalizations (0.2% versus 0.1% OR = 2.6; 95% CI: 1.7–3.9, *p* < 0.001 and 0.5% versus 0.2% OR 3.41; 95% CI: 1.7–6.5, *p* < 0.001, respectively). In a Cox proportional hazards model, PTD was independently associated with long-term maternal renal-related hospitalizations.

Similar to other obstetric complications, it is unknown whether the premature delivery is the cause of later-life maternal morbidity or if it is the result of common predisposing factors that cause premature deliveries in these women. Immune or vascular abnormalities may be such factors. It is known that disorders of deep placentation and pathologic transformation of the spiral arteries are present in a wide range of pregnancy complications. In 2011 Romero et al. reported that placental-bed biopsies in women with placental syndrome showed failure of physiologic transformation of the spiral arteries [67]. Specifically, the mean percentage of spiral arteries that failed physiologic transformation during early pregnancy was significantly higher in women who experienced preterm labor or preeclampsia compared with women with uncomplicated term pregnancies. They proposed that changes in the population and function of immunocytes at the maternal-fetal interface can be part of the mechanism of disease of obstetric disorders.

A history of preterm delivery identifies women who should be targeted for CVD screening and preventative therapies. Future research is needed to assess the potential impact of such preventive measures on the incidence of CVD in this population.

## 5. Other Obstetric Complications

It is evident now that many additional obstetric complications associated with placental pathology result in an increased risk of long-term maternal morbidity. For example, placental abruption, a condition associated with microvascular disturbance, was found to be associated with long term consequences for the mother’s health. For more than 10 years, Pariente et al. followed 653 women who experienced placental abruption and reported that placental abruption was a significant risk factor for long-term cardiovascular mortality [68]. Compared with 46,932 women who delivered during the same period, the cardiovascular case fatality rate for the placental abruption group was 13.0% vs. 2.5% (*p* < 0.001). Women with history of placental abruption had a significantly higher risk for cardiovascular mortality during the follow-up period (Log-rank test *p* = 0.017). Using Cox multivariable regression models, placental abruption remained an independent risk factor for long-term maternal cardiovascular mortality (adjusted hazard ratio (HR) = 4.3; 95% CI 1.1, 18.6). DeRoo et al. explored the risk of CVD-related mortality among women with history of placental abruption among over two million women with a first singleton birth between 1967 and 2002 in Norway, and between 1973 and 2003 in Sweden [69]. Women were followed through 2009 and 2010, respectively, to ascertain subsequent pregnancies and mortality. Cox regression analysis was used to estimate associations between placental abruption and cardiovascular mortality, adjusting for maternal age, education, year of pregnancy and country. There were 49,944 deaths after an average follow-up of 23 years, of which 5453 were due to CVD. Women with placental abruption in first pregnancy (*n* = 10,981) had an increased risk of CVD death (hazard ratio 1.8; 95 % confidence interval 1.3, 2.4). Results were essentially unchanged by excluding women with pre-gestational hypertension, preeclampsia or diabetes. Women with placental abruption in any pregnancy (*n* = 23,529) also had a 1.8-fold increased risk of CVD mortality (95% confidence interval 1.5, 2.2) compared with women who never experienced the condition. Recently, Ananth et al. ascertained CVD events in a cohort of 828,289 women who delivered singletons in Denmark between 1978 and 2010 [70]. With 13,231,562 person-years of follow-up of women with at least one delivery, 11,829 pregnancies were complicated by abruption. The median follow-up in the non-abruption and abruption groups was 16 and 18 years, respectively. CVD mortality rates in women with and without abruption were 0.9 and 0.3 per 10,000 person-years, respectively (adjusted hazard ratio (HR) 2.7, 95% confidence interval (CI) 1.5, 5.0). The corresponding CVD morbidity complication rates were 16.7 and 10.0 per 10,000 person-years, respectively (HR 1.5, 95% CI 1.4, 1.8). The increased risks were evident for ischemic heart disease, acute myocardial infarction, hypertensive heart disease, non-rheumatic valvular disease, and congestive heart failure. On the other hand, Arazi et al. reported that placental abruption was not associated with an increased risk of long-term renal morbidity [71]. Of 99,354 deliveries that occurred during a 25-year period, with a mean follow-up duration of 11.2 years, 1807 (1.8%) had placental abruption. Placental abruption, even though considered a part of the “placental syndrome” with possible vascular etiology, was not found to be a risk factor for long-term maternal renal complications.

Stillbirth and recurrent miscarriages may also be the result of placental pathology. Pariente et al. compared the incidence of long-term atherosclerotic morbidity in a cohort of women with history of stillbirth [72]. Of 99,280 deliveries that met the inclusion criteria, 1879 deliveries (1.9%) were in women who had had at least one stillbirth. After stillbirth, women had a significantly higher cumulative incidence of cardiovascular and renal morbidity and cardiovascular and renal hospitalizations, and had higher rates of simple and complex cardiovascular events. A significant stepwise increase was found between the number of stillbirths and future risk for cardiovascular morbidity. In a Cox proportional hazards model that was adjusted for confounders, previous stillbirth was associated independently with atherosclerotic morbidity. Kessous et al. studied the incidence of atherosclerosis in women who suffered recurrent pregnancy losses (RPL) [73]. They included 99,285 women; 6690 (6.7%) had a history of RPL. Women with history of RPL had higher rates of renal and cardiovascular morbidity, including cardiac invasive and noninvasive diagnostic procedures, simple as well as complex cardiovascular events, and hospitalizations due to cardiovascular causes. Using Kaplan-Meier survival curves, patients with a previous diagnosis of RPL had a significantly higher cumulative incidence of cardiovascular, but not renal hospitalizations. Using a Cox proportional hazards model, adjusted for confounders such as preeclampsia, diabetes mellitus, obesity, and smoking, a history of RPL remained independently associated with cardiovascular hospitalizations.

As expected, maternal obesity during pregnancy is also associated with an increased risk of various long-term maternal morbidity. For example, Sasson et al. reported that obesity during pregnancy is an independent risk factor for long-term ophthalmic complications, and specifically diabetic retinopathy [74]. Moll et al. reported that a high BMI at the beginning of pregnancy significantly increased the risk of several diseases later in life. However, a high weight gain during pregnancy was only significant for future overweight and obesity [75]. The good news is that intervention appears to be beneficial in these situations. In 2016 the Action for Health in Diabetes (Look AHEAD) Study Group reported that in overweight/obese individuals with type 2 diabetes, larger weight losses produced greater improvements in HbA1c, systolic blood pressure, HDL cholesterol, and triglycerides at years one and four (*p* ≤ 0.02) [76]. Even short-term weight loss decreases the risk of cardiovascular disease (CVD). Dodd et al. studied the effect of providing antenatal dietary and lifestyle advice to women who were overweight or obese on components of maternal diet and physical activity and reported that antenatal lifestyle advice improved maternal diet and physical activity during pregnancy [77]. In a study of the effect of lifestyle intervention on markers of maternal metabolism and inflammation in obese women, Renault et al. reported that they were able to reduce a marker of inflammation during pregnancy [78]. In women who are unable to lose weight after pregnancy, bariatric surgery may be a reasonable solution. Purnell et al. reported that diabetes remission up to three years after gastric bypass and gastric banding was proportionally higher with increasing postsurgical weight loss [79].

Obstetric complications are associated with long term risks of complications in the offspring. As mentioned previously, plentiful evidence links low birth weight due to intrauterine growth restriction and increased risk of vascular disease in later adult life. This is considered to be partly the result of programming through fetal nutrition [80]. Delivery of a small for gestational age (SGA) child appears to be a risk factor for future maternal health as well. Pariente at al investigated whether delivering an SGA newborn was a risk factor for subsequent long-term maternal cardiovascular morbidity [81]. Data were analyzed from consecutive pregnant women who delivered between 1988 and 1999, and followed up retrospectively until 2010. Long-term cardiovascular morbidity was compared among women with and without SGA neonates. Of 47,612 deliveries that met the inclusion criteria, 4411 (9.3%) women delivered SGA neonates. Delivery of an SGA neonate was a risk factor for long-term complex cardiovascular events, including congestive heart failure, hypertensive heart and kidney disease, and acute cor pulmonale (odds ratio [OR], 2.3; 95% confidence interval [CI], 1.3–4.4; *p* = 0.006); and long-term cardiovascular mortality (OR, 3.4; 95% CI, 1.5–7.6; *p* = 0.006). Women who delivered an SGA neonate had a significantly higher risk for cardiovascular mortality during the follow-up period (Kaplan-Meier survival analysis, *p* = 0.002). Delivery of an SGA neonate remained an independent risk factor for long-term maternal cardiovascular mortality (Cox multivariable regression: adjusted hazard ratio, 3.5; 95% CI, 1.5–8.2; *p* = 0.004). Almasi et al. studied 99,342 deliveries; 10,701 (10.7%) in women who had at least one previous delivery of an SGA neonate, and reported that delivery of an SGA neonate was an independent risk factor for long-term maternal renal disease [82].

## 6. Conclusions

It is clear that many obstetric complications are associated with increased risk of long-term maternal morbidity. It is likely that some, if not all, are due to common predisposing factors in these women. To improve women’s health and decrease such risks, both women themselves and the medical team caring for them need to be aware of these risks. Multiple interventions including diet modifications, weight loss, and increased physical activity appear to be effective in decreasing these risks. For a variety of reasons, women should be encouraged to breastfeed. Regarding prevention of long-term morbidity, Peters et al. reported that giving birth to more children was found to be associated with a higher risk of CHD later in life, whereas breastfeeding was associated with a lower CHD risk; women who both had children and breastfed had a non-significantly higher risk of CHD [83]. Dancing is an enjoyable activity that was also reported to have a risk-reducing effect [84]. Moderate-intensity dancing was associated with reduced risk of cardiovascular disease mortality to a greater extent than walking. The association between dance and cardiovascular disease mortality may be explained by high-intensity bouts during dancing, lifelong adherence, or psychosocial benefits.
